# CpG Oligodeoxynucleotides Modulate Innate and Adaptive Functions of IgM^+^ B Cells in Rainbow Trout

**DOI:** 10.3389/fimmu.2019.00584

**Published:** 2019-03-26

**Authors:** Rocío Simón, Patricia Díaz-Rosales, Esther Morel, Diana Martín, Aitor G. Granja, Carolina Tafalla

**Affiliations:** Fish Immunology and Pathology Laboratory, Animal Health Research Center (CISA-INIA), Madrid, Spain

**Keywords:** teleost fish, rainbow trout, CpGs, B cells, IgM, phagocytosis

## Abstract

Oligodeoxynucleotides (ODN) containing unmethylated CpG motifs have been widely postulated as vaccine adjuvants both in mammals and teleost fish. However, to date, the effects that CpGs provoke on cells of the adaptive immune system remain mostly unexplored in fish. Given that rainbow trout (*Oncorhynchus mykiss*) IgM^+^ B cells from spleen and blood transcribe high levels of toll like receptor 9 (TLR9), the receptor responsible for CpG detection in mammals, in the current work, we have investigated the effects of CpGs on both spleen and blood IgM^+^ B cells from this species. CpGs were shown to exert strong proliferative effects on both spleen and blood IgM^+^ B cells, also increasing their survival. The fact that CpGs increase the size of IgM^+^ B cells, reduce the expression of surface IgM and IgD and up-regulate the number of IgM-secreting cells strongly suggest that IgM^+^ B cells differentiate to plasmablasts/plasma cells in response to CpG stimulation. Additionally, CpGs were shown to modulate the antigen presenting capacities of trout IgM^+^ B cells through an increased surface MHC II expression and transcriptional up-regulation of co-stimulatory molecules, although in this case, significant differences were observed between the effects exerted on spleen and blood cells. Similarly, differences were observed between spleen and blood IgM^+^ B cells when CpG stimulation was combined with B cell receptor (BCR) cross-linking. Finally, CpGs were also shown to affect innate functions of teleost IgM^+^ B cells such as their phagocytic capacity. These results demonstrate that CpGs regulate many adaptive and innate functions of teleost B cells, supporting their inclusion as adjuvants in novel vaccine formulations.

## Introduction

At the initial phases of an infection, cells of the innate immune system detect pathogens through the recognition of common pathogen-associated molecular patterns (PAMPs) by germline-encoded pattern recognition receptors (PRRs). Among these PRRs, Toll-like receptors (TLRs) constitute a large family of PRRs, expressed either on the cell surface or the luminal side of intracellular vesicles such as endosomes or lysosomes. These receptors are capable of detecting a wide range of pathogen-associated molecules, such as unmethylated DNA, peptidoglycan, dsRNA, ssRNA, or bacterial lipopolysaccharide (LPS) among others (1).

Interestingly, B cells also express a variable range of TLRs that allow them to directly respond to microbial products, in addition to a clonally-rearranged B cell receptor (BCR) that will respond to specific antigens. Thus, this dual expression program provides B cells with the exclusive machinery to integrate at the same time antigen-specific and innate signals (2). However, in mammals, not all B cell subsets express all TLRs and therefore how each of these subsets responds to TLR ligands differs considerably. For example, in humans, naïve B cells express very low levels of TLRs whereas memory B cells constitutively express a wide range of TLRs through which they regulate their proliferation and differentiation (3). In mice, it has been shown that different subsets of B cells express a qualitatively similar but quantitatively different pattern of TLR transcripts, consequently responding differently to stimulation with TLR agonists (4).

In teleost fish, three main subsets of B cells are found in homeostasis. The main subset corresponds to B cells that co-express IgM and IgD on the cell surface (IgM^+^ B cells) (5). These cells are found in lymphoid organs (spleen and head kidney), circulating blood, liver, adipose tissue and in mucosal surfaces (6, 7). Remarkably, these cells differ greatly in many aspects with mammalian conventional B2 cells, as they have been shown to share several phenotypic and functional traits of mammalian innate B1 cell populations (5). Additionally, cells that exclusively express IgD on the surface (IgD^+^ cells) have been identified in rainbow trout (*Oncorhynchus mykiss*) gills (8) and catfish (*Ictalurus punctatus*) blood (9). Finally, an independent B cell linage that exclusively expresses IgT on the surface (IgT^+^ cells) is also present in most fish species (10). IgT is a teleost-exclusive Ig and in the absence of IgA, IgT^+^ B cells have been postulated as lymphocytes specialized in mucosal immunity based on the fact that the ratio of IgT^+^ B cells to IgM^+^ B cells is higher in mucosal surfaces, and because IgT responses to several parasites seemed to be confined to mucosal compartments (10–12).

To date, in teleost, the expression of TLRs has only been studied in IgM^+^ B cells. Thus, in rainbow trout, IgM^+^ B cells from different systemic and mucosal tissues were shown to transcribe all TLRs identified at that moment in this species (TLR1, TLR2, TLR3, TLR5, TLR7, TLR8, TLR9, and TLR22) (6). In Atlantic salmon (*Salmo salar*), IgM^+^ B cells from lymphoid tissues were also shown to transcribe TLR3, TLR9, TLR8a1, TLR21, and TLR22 (13). Among these TLRs, mammalian TLR9 is responsible for the recognition of foreign DNA molecules from bacteria or viruses that contain short sequences of unmethylated CpG dinucleotides (14). The capacity of teleost TLR9 to respond to CpGs has been suggested based mainly on indirect transcriptional studies, however additional research is still required to unequivocally confirm that TLR9 is sensing CpG in these species (15). Based on structural characteristics, three classes of CpGs exist (A, B, and C), all of them with the capacity to stimulate TLR9, but with important differences in the effects they exert on different leukocyte subpopulations (16). In any case, the expression of TLR9 in teleost IgM^+^ B cells seems to predict a responsiveness of these cells to CpGs, already demonstrated in Atlantic salmon by Jenberie et al. (13). In that study, it was revealed that the incubation of leukocyte cultures with CpGs up-regulated the transcription of secreted IgM, CD83 and CD40 in IgM^+^ B cells. Furthermore, an up-regulation of IgM and MHC II protein levels in sorted IgM^+^ B cells was also demonstrated by Western blot (13).

As mentioned above, in mammals, different B cell subsets respond differently to TLR ligation and these differences are also visible in what concerns TLR9 stimulation. Thus, in mice, although follicular B2 cells proliferate to a higher extent in response to CpGs than B1 cell subsets, only B1 and marginal zone (MZ) cells differentiate to plasma cells in response to TLR9 stimulation in the absence of BCR engagement (4). On the other hand, when BCR cross-linking is combined with TLR9 stimulation, murine B2 cells are able to proliferate and differentiate into class-switched plasma cells both *in vitro* and *in vivo* (17). In humans, on the other hand, although naïve B cells have the capacity to proliferate in response to CpGs alone (18), it is IgM^+^ memory B cells that are much more responsive to TLR9 stimulation in the absence of BCR engagement (19).

In this context, in the current work, we have expanded our knowledge on how teleost IgM^+^ B cells respond to CpGs, by studying the effects of CpGs on a wide range of functions of rainbow trout IgM^+^ B cells, including proliferation and survival, IgM secretion, surface expression of Igs and MHC II, phagocytic capacity, and responsiveness to BCR cross-linking. We have performed this study with both splenic and blood IgM^+^ B cells, observing important differences in the way that these two cell subsets respond to CpGs. Given that CpGs have been postulated as possible adjuvants to be included in newly designed vaccination strategies for aquacultured fish, our results provide highly valuable information on the capacity that these molecules have to stimulate both innate and adaptive functions of teleost B cells.

## Materials and Methods

### Experimental Fish

Healthy specimens of female rainbow trout (*Oncorhynchus mykiss*) of ~50–70 g were obtained from Centro de Acuicultura El Molino (Madrid, Spain) and maintained at the animal facilities of the Animal Health Research Center (CISA-INIA, Instituto Nacional de Investigación y Tecnología Agraria y Alimentaria) in an aerated recirculating water system at 16°C with 12:12 h light/dark photoperiod. Fish were fed twice a day with a commercial diet (Skretting, Spain). Before any experimental procedure, fish were acclimatized to laboratory conditions for at least 2 weeks. All of the experiments described comply with the Guidelines of the European Union Council (2010/63/EU) for use of laboratory animals and have been approved by the INIA Ethics committee (Code CEEA PROEX002/17).

### Tissue Sampling

Rainbow trout were killed by benzocaine (Sigma) overdose and blood and spleen collected. Blood was extracted with a heparinized needle from the caudal vein and diluted 10 times with Leibovitz medium (L-15, Thermo Fisher Scientific) supplemented with 100 IU/ml penicillin and 100 μg/ml streptomycin (P/S, Thermo Fisher Scientific), 5% fetal calf serum (FCS, Thermo Fisher Scientific) and 10 IU/ml heparin (Sigma). Spleen was collected and single cell suspensions were obtained using 100 μm nylon cell strainer (BD Biosciences) and L-15 medium supplemented with antibiotics, 5% FCS and heparin. Blood cell suspensions were placed onto 51% Percoll (GE Healthcare) cushions whereas spleen suspensions were placed onto 30/51% discontinuous Percoll density gradients. All gradients were then centrifuged at 500 × *g* for 30 min at 4°C. The interface cells were collected, washed twice in L-15 containing antibiotics and 5% FCS and adjusted to 2 × 10^6^ cells/ml.

### Cell Stimulation

Total leukocyte populations from spleen or blood were cultured at 20°C in L-15 medium supplemented with antibiotics and 5% FCS in 24 or 96-well plates (Nunc). Different stimuli were added to the media and cells were incubated for different time periods depending on specific experiments. The phosphorothioate-modified B class CpG oligodeoxynucleotide (ODN) 1668 (InvivoGen) containing one CpG dinucleotide (CpG) (5′-tccatgaCGttcctgatgct-3′) was used at a final concentration of 5 μM after having determined the optimal concentration based on their positive effect on B cell survival, specifically choosing the concentration that provoked the higher B cell survival after 72 h of incubation (data not shown). The non-CpG ODN 1668 (that contains GpC dinucleotides instead of CpGs) (5′-tccatgaGCttcctgatgct-3′) was used as a negative control (non-CpG) at the same concentration. In some experiments, leukocytes were stimulated with an unlabeled monoclonal antibody (mAb) against trout IgM (clone 1.14, mouse IgG1) (20) at a final concentration of 10 μg/ml as previously described (5). Non-stimulated controls were always included.

### B Cell Proliferation

The Click-iT Plus EdU Flow Cytometry Assay Kit (Sigma) was used to measure the proliferation of IgM^+^ B cells following manufacturer's instructions. Briefly, blood and spleen leukocyte suspensions at a concentration of 2 × 10^6^ cells per ml were incubated in 96-well plates for 3 days at 20°C with different stimuli depending on the specific experiment as described above. Thereafter, 5-ethynyl-2′-deoxyuridine (EdU) was added to the cultures at a final concentration of 1 μM and the cells were incubated for an additional 24 h. After that time, stimulated and unstimulated cells were collected and stained with anti-IgM (1.14) coupled to allophycocyanin (1 μg/ml) for 20 min at 4°C. Whenever cells had been stimulated with anti-IgM, the cells were only labeled with EdU (1 μM) as described above. The incorporation of EdU to the DNA was determined following the manufacturer's instructions and then analyzed by flow cytometry in a FACS Calibur flow cytometer (BD Biosciences) equipped with CellQuest Pro software (BD Biosciences). Flow cytometry analysis was performed with FlowJo V10 (TreeStar).

### ELISPOT Analysis

ELISPOT plates containing Inmobilon-P membranes (Millipore) were activated with 70% ethanol for 30 s, coated with an anti-IgM mAb (clone 4C10) at 2 μg/ml in phosphate buffer saline (PBS) and incubated overnight at 4°C. To block non-specific binding to the membrane, plates were then incubated with 2% bovine serum albumin (BSA) in PBS for 2 h at RT. Leukocyte suspensions from spleen or blood of individual fish that had been stimulated with CpG or non-CpG at 5 μM for 72 h at 20°C or left unstimulated in the same conditions were then added to the wells in triplicate at a concentration of 5 × 10^4^ cells per well. After 24 h of incubation at 20°C, cells were washed away five times with PBS and plates blocked again with 2% BSA in PBS for 1 h at RT. After blocking, biotinylated anti-IgM mAb (clone 4C10) was added to the plates and incubated at 1 μg/ml for 1 h at RT. Following additional washing steps (five times in PBS), the plates were developed using streptavidin-HRP (Thermo Scientific) at RT for 1 h, washed again with PBS and incubated with 3-amino-9-ethylcarbazole (Sigma Aldrich) for 30 min at RT in the dark. Substrate reaction was stopped by washing the plates with water. Once the membranes had dried, they were digitally scanned and the number of spots in each well-determined using an AID iSpot Reader System (Autoimmun Diagnostika GMBH).

### Flow Cytometry

Blood or spleen leukocytes seeded in 96-well plates at a density of 2 × 10^6^ cells per ml were incubated for 72 h at 20°C with 5 μM CpG, 5 μM non-CpG or media alone. After the incubation period, cells were washed in staining buffer (PBS containing 1% FCS and 0.5% sodium azide) and co-incubated with FITC-conjugated anti-IgM (1.14) (1 μg/ml) and specific mAbs against trout MHC II β-chain (mAb mouse IgG_1_ coupled to allophycocyanin, 2 μg/ml) or trout IgD (mAb mouse IgG_1_ coupled to allophycocyanin, 10 μg/ml) previously characterized (21, 22). Thereafter, cells were washed twice with the same buffer and analyzed in a FACS Calibur flow cytometer equipped with CellQuest Pro software. Flow cytometry analysis was performed with FlowJo V10. In these cultures, cell survival was estimated determining the percentage of live IgM^+^ B cells in the cultures after counterstaining the cells with 1 μg of Propidium iodide (Invitrogen).

### Cell Sorting

IgM^+^ B cells were isolated from blood and spleen leukocyte cultures by flow cytometry using a BD FACSAria III cell sorter (BD Biosciences). For this purpose, spleen and blood leukocytes were first seeded in 24-well plates at a density of 2 × 10^6^ cells per ml and incubated for 24 h at 20°C with 5 μM CpG, 5 μM non-CpG, or media alone. After that time, cells were collected and incubated for 20 min at 4°C with an anti-IgM (1.14) mAb coupled to allophycocyanin in staining buffer. Following two washing steps, cells were resuspended in staining buffer and IgM^+^ B cells isolated based on their FSC/SSC profiles (to exclude the granulocyte gate) and then on the basis of the fluorescence emitted by the anti-IgM antibody coupled to allophycocyanin. Approximately 70.000 IgM^+^ B cells and the same amount of IgM^−^ cells were collected in PBS for subsequent RNA isolation.

To confirm a direct effect of CpGs on IgM^+^ B cells, spleen and blood leukocytes were incubated for 20 min at 4°C with a biotinilated Fab fragment of anti-IgM 1.14 (to avoid cell activation) in staining buffer. Following two washing steps, Streptavidin-Phycoerythrin (PE) (BD Pharmingen) was added. After 20 min at 4°C, cells were resuspended in staining buffer and IgM^+^ B cells isolated as described above. Sorted IgM^+^ B cells were then incubated with 5 μM CpG, 5 μM non-CpG, or media alone for 3 days at 20°C. After this time, cells were stained with anti-MHC II mAb coupled to allophycocyanin, counterstained with 0.2 μg/ml DAPI, and analyzed on a FACS Celesta flow cytometer (BD Biosciences).

### Real Time PCR Analysis

Total RNA was isolated from FACS isolated IgM^+^ B cell populations using the Power SYBR Green Cells-to-Ct Kit (Invitrogen) following the manufacturer's instructions. RNA was treated with DNase during the process to remove genomic DNA that might interfere with the PCR reactions. Reverse transcription was also performed using the Power SYBR Green Cells-to-Ct Kit following the manufacturer's instructions. To evaluate the levels of transcription of the different genes, real-time PCR was performed with a LightCycler® 96 System instrument (Roche) using SYBR Green PCR core Reagents (Applied Biosystems) and specific primers previously described (Table S1). Samples obtained from individual fish were analyzed in duplicate under the following conditions: 10 min at 95°C, followed by 40 amplification cycles (15 s at 95°C and 1 min at 60°C). A melting curve for each primer set was obtained by reading fluorescence every degree between 60 and 95°C to ensure that only a single PCR product had been amplified. The expression of individual genes was normalized to the relative expression of the housekeeping gene β-actin, and the expression levels were calculated using the 2-ΔCt method, where ΔCt is determined by subtracting the actin value from the target Ct (Ct cut-off set to 38). β-actin was selected as reference gene according to the MIQE guidelines (23) given that no statistical differences were detected among Ct values obtained for β-actin in the different samples. In any case, all results were confirmed using another reference gene, elongation factor 1α (EF-1α). Negative controls with no template and *minus* reverse transcriptase controls were included in all the experiments.

### Phagocytic Activity

To analyze the effect of CpGs on the phagocytic capacity of spleen and blood IgM^+^ B cells, spleen and blood leukocytes were seeded in 24-well plates at a cell density of 2 × 10^6^ cells per well and incubated for 48 h at 20°C with the appropriate stimuli (5 μM CpG, 5 μM non-CpG, or media alone). After 48 h, the cells were collected and resuspended in L-15 medium without serum. The cells were then incubated for 3 h at 20°C with fluorescent beads (FluoSpheres® Microspheres, 1.0 μm, Crimson Red Fluorescent 625/645, 2% solids; Thermo Fisher Scientific) at a cell:bead ratio of 1:10 as described before (24). After the incubation period, cells were harvested by gently pipetting, and non-ingested beads were removed by centrifugation (100 × *g* for 10 min at 4°C) over a cushion of 3% (weight/volume) BSA (Fraction V; Fisher Scientific) in PBS supplemented with 4.5% (weight/volume) D-glucose (Sigma). Cells were then resuspended in staining buffer, labeled with anti-IgM-FITC (1.14) (1 μg/ml) and analyzed on a FACS Calibur flow cytometer. In some experiments, cytochalasin B (0.05 μg/ml) was added to the cells immediately before the addition of the beads to verify active phagocytosis. Flow cytometry analysis was performed with FlowJo V10 software.

### Statistical Analysis

Statistical analyses were performed to compare values obtained in each experimental group using a two-tailed paired Student's *t* test with Welch's correction when the F test indicated that the variances of both groups differed significantly. The differences between the mean values were considered significant on different degrees, where ^*^ means *p* ≤ 0.05, ^**^ means *p* ≤ 0.01, and ^***^ means *p* ≤ 0.005.

## Results

### CpGs Induce IgM^+^ B Cell Proliferation and Survival

**Figure 1:**
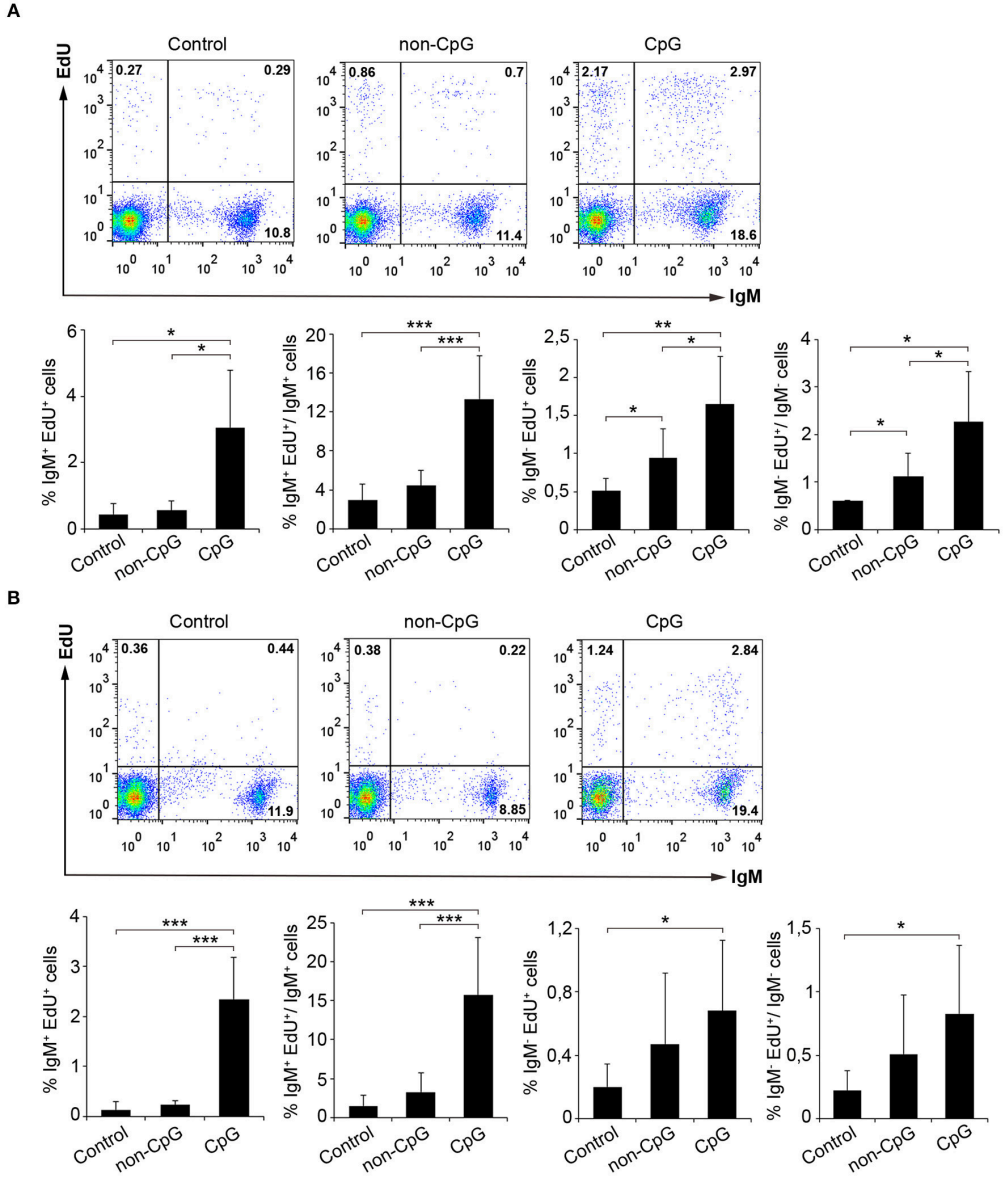
CpGs induce B cell proliferation. The lymphoproliferative effects of CpGs were determined incubating spleen **(A)** or blood **(B)** leukocytes with CpG (5 μM), non-CpG (5 μM) or media alone (Control) during 3 days at 20°C. After this time, cells were labeled with EdU (1 μM) and incubated for a further 24 h. At that point, cells were labeled with anti-IgM mAb and the number of IgM^+^ B cells with incorporated EdU (proliferating cells) determined as described in section Materials and Methods. Representative dot plots are included along with graphs showing the quantification of proliferating IgM^+^ and IgM^−^ cells among total leukocytes or the corresponding IgM^+^ or IgM^−^ populations (mean + SD) (*n* = 6 fish). Asterisks denote significantly different values between indicated groups (^*^p ≤ 0.05, ^**^p ≤ 0.01, and ^***^p ≤ 0.005).

In mammals, type B CpGs are particularly efficient in promoting the proliferation and survival of naïve B cells in comparison to other stimuli such as poly I:C, LPS, or flagellin (25). Thus, we analyzed the lymphoproliferative effect of CpG ODNs (type B) in both rainbow trout splenocytes and blood leukocytes. Results obtained from these *in vitro* experiments clearly showed that rainbow trout splenic and blood IgM^+^ B cells significantly proliferated in response to CpG treatment in comparison to the proliferation rates obtained in untreated cultures (Control) or cultures treated with non-CpG ODNs (non-CpG) (Figures 1A,B). Thus, both in spleen and in blood, CpGs increased both the percentage of proliferating IgM^+^ B cells in relation to the total leukocyte population, as well as the percentage of proliferating IgM^+^ B cells within the IgM^+^ B cell compartment (Figures 1A,B). The fact that non-CpG ODNs did not have a stimulatory effect on lymphocyte proliferation rates strongly suggests that the lymphoproliferative effects exerted by CpGs on IgM^+^ B cells are mediated through a CpG-specific TLR signaling. Interestingly, CpGs not only increased the percentage of proliferating IgM^+^ B cells in the cultures (IgM^+^EdU^+^ cells) but also the percentage of IgM^+^ B cells that were not proliferating (IgM^+^EdU^−^ cells) (Figures 1A,B), suggesting a positive effect of CpGs on IgM^+^ B cell survival, independent of proliferation. The lymphoproliferative effects exerted by CpGs were not only visible on IgM^+^ B cells, since IgM^−^ cells also proliferated significantly in response to CpGs (Figures 1A,B). However, in the case of IgM^−^ cells, non-CpG ODNs were also capable of provoking some degree of proliferation (Figures 1A,B), suggesting a less specific effect on these cells. In fact, in blood, IgM^−^ cells proliferated in response to CpGs at rates that were not significantly different than those observed in response to non-CpG ODNs (Figure 1B).

### CpGs Down-Regulate the Expression of IgM and IgD on the Surface of Rainbow Trout B Cells

**Figure 2:**
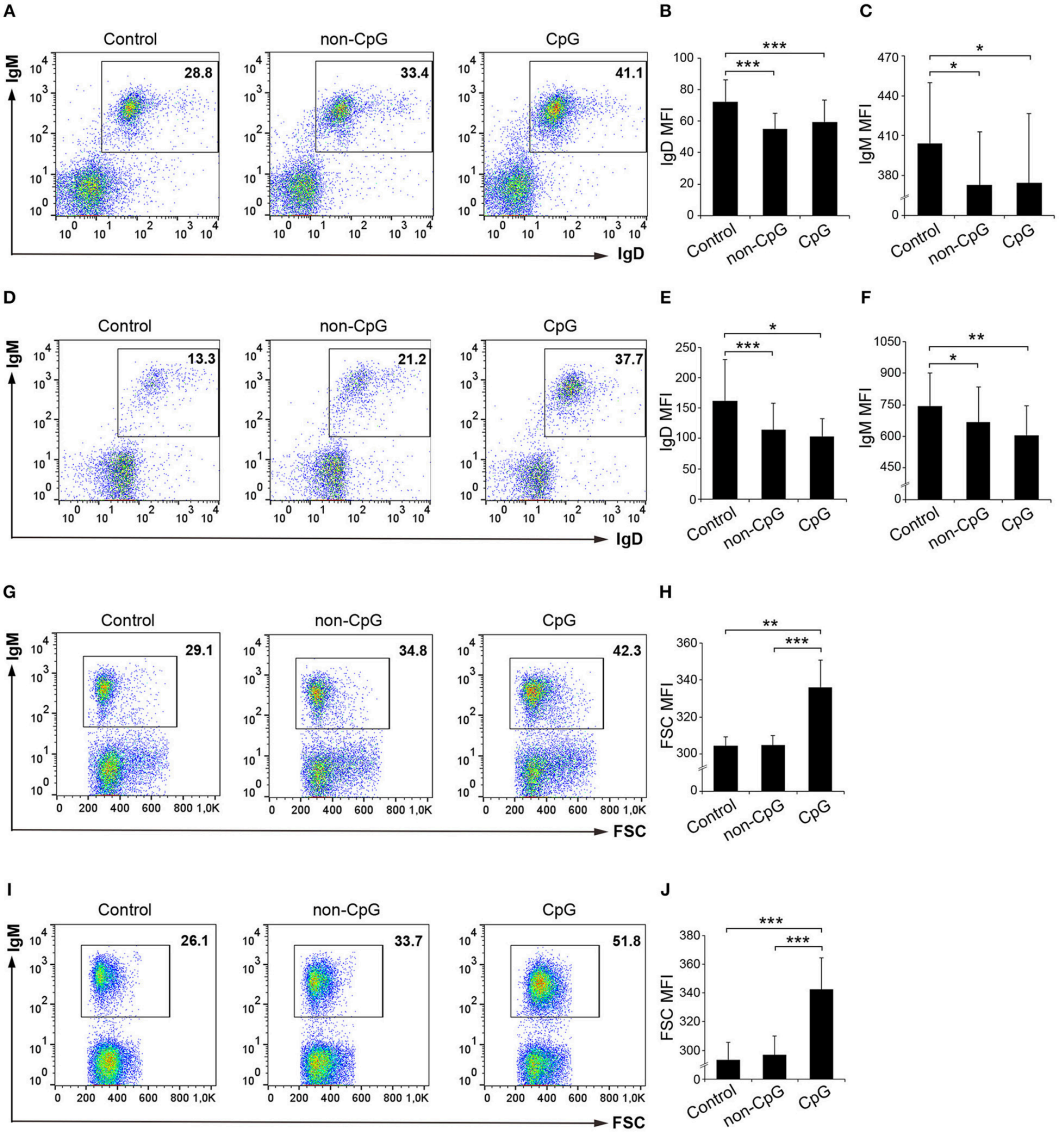
Effect of CpG treatment on IgM and IgD surface expression and size. Spleen and blood leukocytes were incubated with CpG (5 μM), non-CpG (5 μM), or media alone (Control) during 3 days at 20°C. The levels of IgD and IgM surface expression on B cells were then measured via flow cytometry using a specific mAbs in spleen **(A–C)** and blood **(D–F)**. Representative dot plots from spleen **(A)** and blood **(B)** leukocytes are included along with graphs showing mean fluorescence intensity (MFI) values for IgD **(B,E)** and IgM **(C,F)** (mean + SD; *n* = 5 fish). In this experiment, the size of the B cells was also calculated. Representative dot plots from spleen **(G)** and blood **(I)** leukocytes showing the Forward scatter (FSC) of IgM^+^ B cells are included along with graphs showing the FSC MFI values **(H,J)** (mean + SD; *n* = 5 fish). Asterisks denote significantly different values between indicated groups (^*^p ≤ 0.05, ^**^p ≤ 0.01, and ^***^ *p* ≤ 0.005).

We next studied whether CpG ODNs affected the expression levels of surface IgM and IgD on rainbow trout splenic as well as blood IgM^+^ B cells. To assess this, we stimulated leukocyte cultures with CpG ODNs, non-CpG ODNs, or media alone for 72 h and afterwards we analyzed the levels of expression of membrane IgD and IgM by flow cytometry. At this point, it was evident that CpGs exerted a positive effect on IgM^+^ B cell survival as the percentage of IgM^+^ B cells in splenic or blood cultures increased 1.5 and 2 fold, respectively, in the presence of CpGs (Figures 2A,D). Furthermore, the levels of IgD and IgM on the cell surface were significantly reduced in IgM^+^ B cells from CpG-treated cultures when compared to untreated cells, both in spleen and in blood (Figures 2A–F). Intriguingly, this effect was also observed when cultures were incubated with non-CpG ODNs (Figures 2A–F). In any case, this reduction of IgM and IgD surface expression could be indicating a differentiation of naïve B cells to plasmablasts/plasma cells given that throughout this differentiation process B cells lose IgD and reduce their IgM expression on the cell surface both in mammals and in fish (26, 27). As it is also known that throughout this differentiation process IgM^+^ B cells also increase in size (26, 27), we also studied the effects of CpG on the size of IgM^+^ B cells. We found a dramatic size increase of spleen and blood IgM^+^ B cells in response to CpGs (Figures 2G–J). In this case, the increase was evident when compared to both untreated IgM^+^ B cells and IgM^+^ B cells from cultures treated with non-CpG ODNs (Figures 2G–J), suggesting a specific effect.

In our general experimental design, total leukocytes populations were incubated with CpGs. Thus, it might have been possible that the effects exerted on IgM^+^ B cells could have been a consequence of an indirect effect by stimulation of another leukocyte subset that secreted cytokines or factors that affected IgM^+^ B cells upon activation. To rule out this possibility, we performed an additional experiment in which IgM^+^ B cells were first sorted and then stimulated with CpGs. In this experiment, we confirmed that IgM^+^ B cells directly sense CpGs. Sorted blood IgM^+^ B cells stimulated with CpGs had a significantly increased survival rate when compared to IgM^+^ B cells incubated with non-CpGs or media alone (Figure S1). Sorted splenic IgM^+^ B cells, on the other hand, when stimulated with CpGs, increased their survival rate in comparison to unstimulated controls but not when compared to non-CpG-treated cultures (Figure S1). Both blood and spleen IgM^+^ B cells significantly increased size in comparison to IgM^+^ B cells incubated with non-CpGs or media alone (Figure S1).

### CpGs Activate IgM Secretion in Naïve B Cells

**Figure 3:**
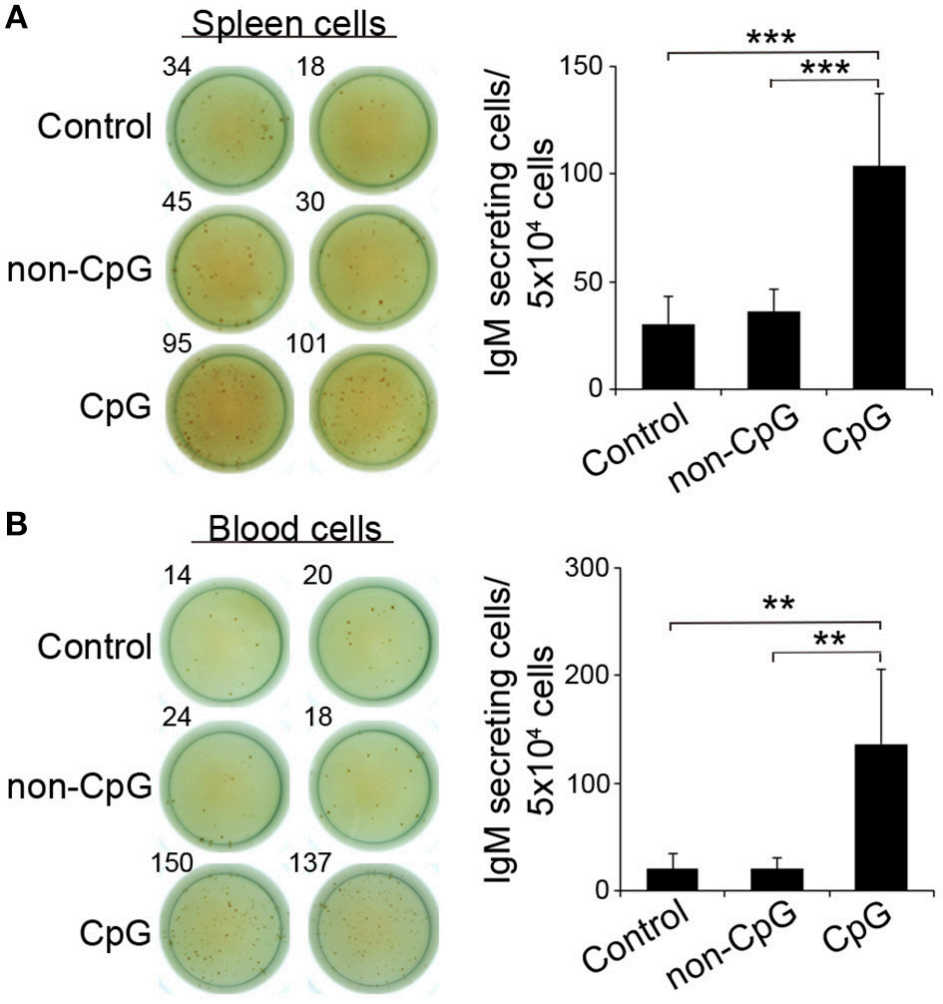
CpGs increase the number of IgM-secreting cells. ELISPOT analysis of IgM-secreting cells in spleen **(A)** and blood **(B)** cultured cells treated with CpG (5 μM), non-CpG (5 μM), or media alone. Cells were cultured with the appropriate stimuli for 48 h and then plated in ELISPOT plates, previously coated with anti-IgM mAb (2 μg/ml) for a further 24 h. After incubation, cells were washed away and a biotinylated anti-IgM mAb (1 μg/ml) was used to detect number of spot forming cells. Duplicates for a representative individual (left) and quantification of spot forming cells (right) from 6 independent fish are shown (mean + SD). Asterisks denote significantly different values between indicated groups (^**^p ≤ 0.01 and ^***^p ≤ 0.005).

In mice, CpGs alone have been shown to induce an increased IgM secretion by promoting the differentiation of innate B1 populations to plasma cells (4). Similarly, in rainbow trout, the incubation of trout spleen and blood leukocytes with CpG ODNs significantly increased the number of IgM-secreting cells after 3 days in comparison to the number of IgM-secreting cells found in non-stimulated or non-CpG treated cultures (non-CpG), as verified in an ELISPOT assay (Figures 3A,B).

### CpGs Up-Regulate the Surface Expression of MHC-II in Naïve Trout B Cells and Induce the Expression of Co-stimulatory Molecules

**Figure 4:**
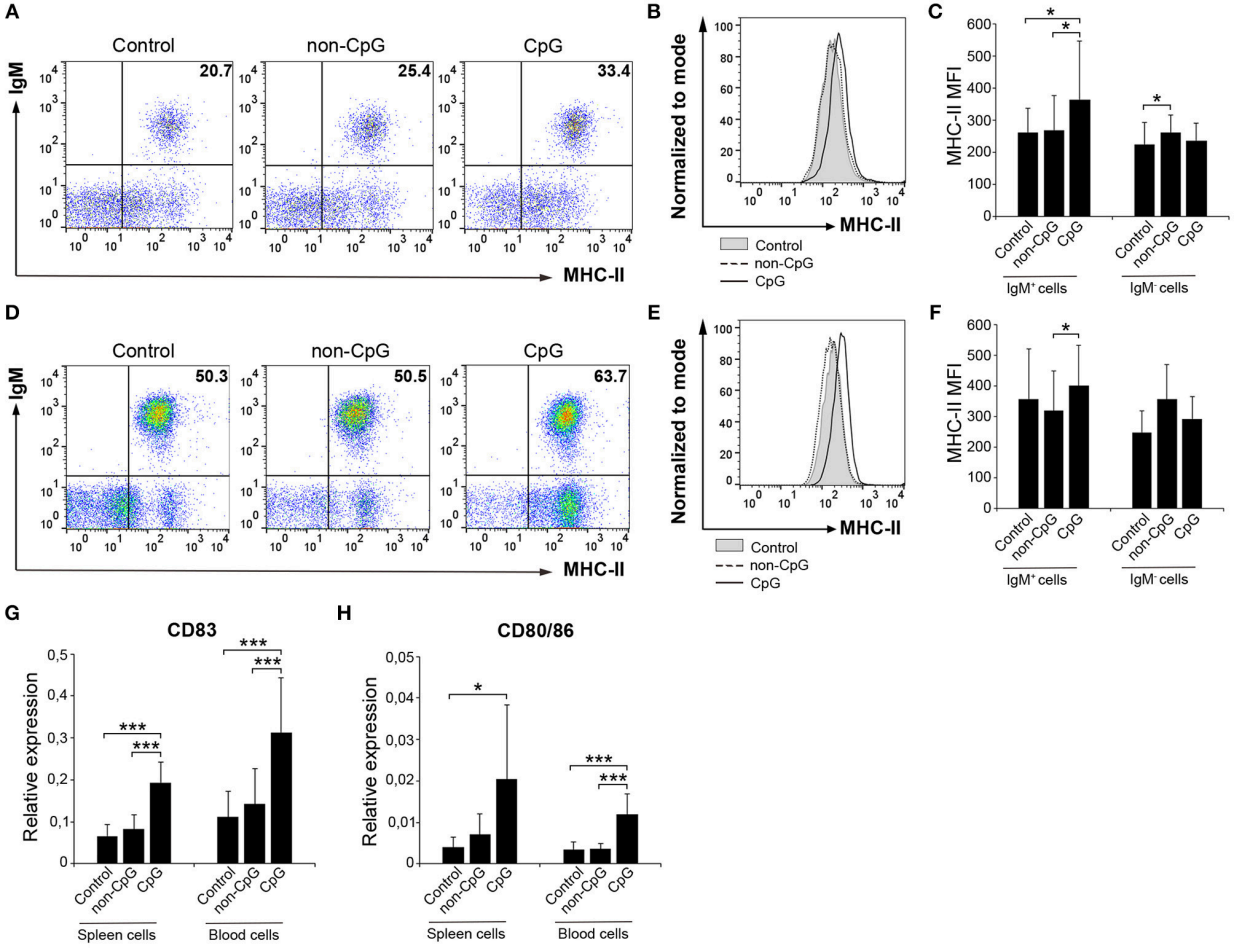
CpGs upregulate MHC-II surface expression and increase the transcription of co-stimulatory molecules. Spleen and blood leukocytes were incubated with CpG (5 μM), non-CpG (5 μM), or media alone (Control) during 3 days at 20°C. The levels of MHC-II expression on the surface of IgM^+^ B cells were then measured via flow cytometry using a specific mAb against trout MHC-II. Representative dot plots from spleen **(A)** and blood **(D)** leukocytes are included along with corresponding histograms **(B,E)**. Graphs showing MHC-II MFI values for IgM^+^ and IgM^−^ cells in spleen **(C)** and blood **(F)** are also shown (mean + SD; *n* = 7 fish). Spleen and blood leukocytes were incubated with CpG (5 μM), non-CpG (5 μM), or media alone (Control) for 24 h at 20°C. IgM^+^ cells were then isolated by flow cytometry and RNA extracted to determine the levels of transcription of CD83 **(G)** and CD80/86 **(H)** co-stimulatory genes. Gene expression data were normalized against the endogenous control gene β-actin and shown as relative expression (mean + SD; *n* = 7–9 fish). Asterisks denote significantly different values between indicated groups (^*^p ≤ 0.05 and ^***^p ≤ 0.005).

B cells are professional antigen-presenting cells (APCs) that constitutively express MHC II on the cell surface (28). Furthermore, fish IgM^+^ B cells, as a consequence of their phagocytic activity, have been postulated as cells with increased presenting capacities than those of mammalian B cells (29). Thus, we also established whether CpGs could affect the expression of MHC II on the surface of rainbow trout splenic and blood IgM^+^ B cells. Our results clearly show that CpGs significantly increased the levels of surface MHC II on IgM^+^ B cells from spleen (Figures 4A–C). Interestingly, this increase in surface MHC II levels was not visible in splenic IgM^−^ cells carrying MHC II on the cell surface (Figure 4C), which in this organ would mainly account for IgT^+^ B cells (10). Furthermore, blood IgM^+^ B cells did not regulate surface MHC II expression in response to CpGs in a significant fashion, given that the values obtained in CpG-treated cultures were not significantly different than those obtained in untreated cultures (Figures 4D–F). Despite the differential effect of CpGs on the levels of surface MHC II expression between the two populations studied when total leukocyte cultures were used, CpGs up-regulated the levels of transcription of co-stimulatory molecules in FACS isolated IgM^+^ B cells from both spleen and blood (Figures 4G,H). Thus, stimulation with CpGs provoked that splenic and blood IgM^+^ B cells upregulated CD83 and CD80/86, a molecule with similar homologies to both mammalian CD80 and CD86 (30) (Figures 4G,H). Furthermore, when sorted IgM^+^ B cells were incubated with CpGs, a significant increase in surface MHC II levels was observed both in splenic and blood IgM^+^ B cells (Figure S1).

### CpGs Induce the Phagocytic Activity of IgM^+^ B Cells

Teleost B cells, similarly to mammalian B1 cell subsets (31), have been shown to have a potent phagocytic activity (32). Therefore, we also investigated if CpG ODNs could have an effect on the capacity of IgM^+^ B cells to phagocytose microparticles. To analyze this aspect, splenocytes and blood cells were incubated with CpG, non-CpG or with media alone for 48 h. After this time, 1 μm Crimson red-labeled polystyrene beads were added to the cultures and after 3 h of incubation the phagocytic activity of IgM^+^ B cells determined through flow cytometry.

**Figure 5:**
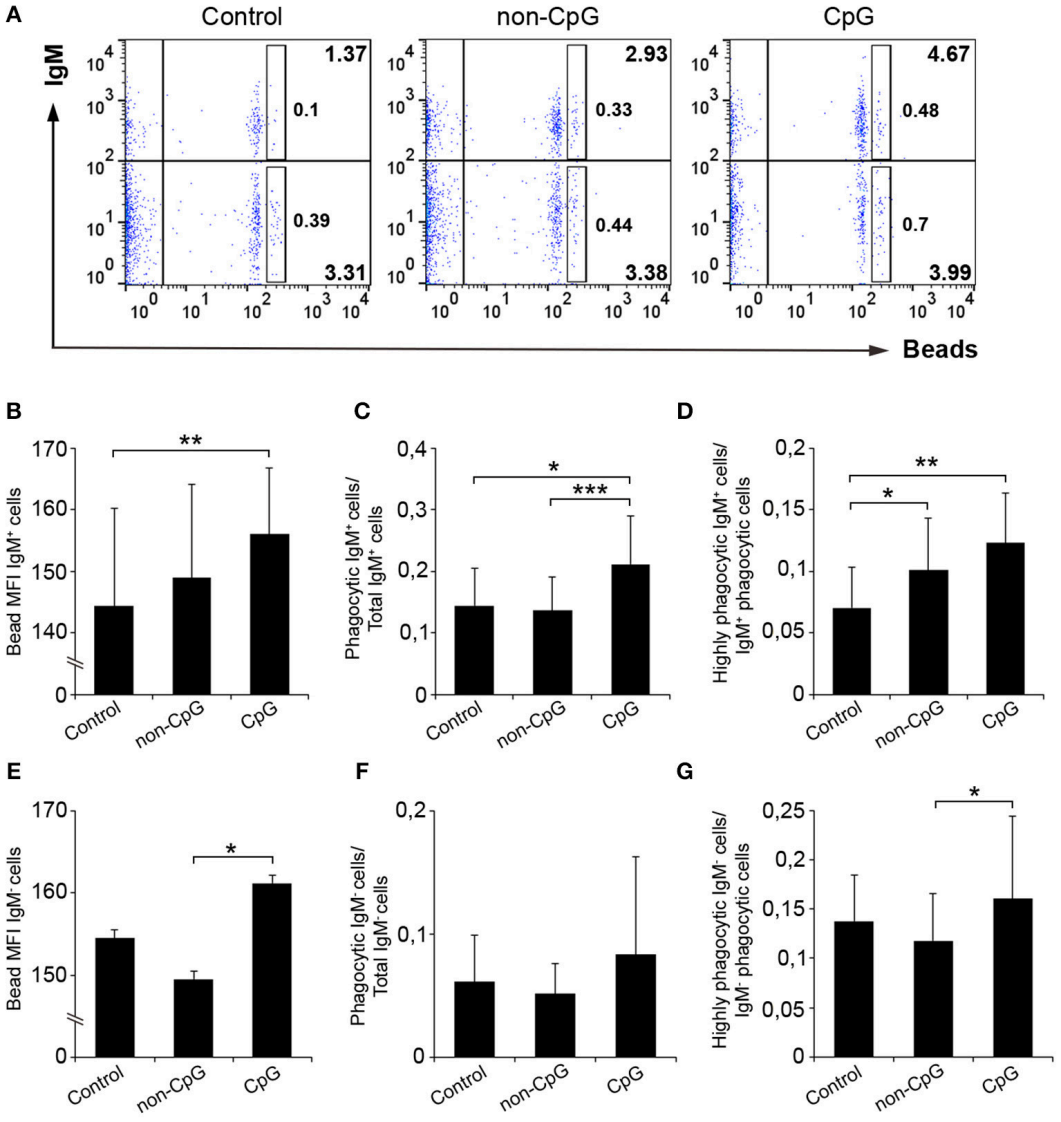
CpGs up-regulate the phagocytic capacities of IgM^+^ B cells from spleen. Splenocytes were cultured in the presence of CpG, non-CpG, or media alone as previously indicated for 48 h at 20°C. After this time, cells were incubated with Crimson Red fluorescent beads (1 μm diameter) at a ratio of 1:10 (cell/beads) for a further 3 h at 20°C. Non-ingested beads were removed by centrifugation over a cushion of 3% BSA supplemented with 4.5% D-glucose. Cells were then stained with anti-IgM mAb and analyzed by flow cytometry. **(A)** Representative dot plots for each experimental condition are shown. The rectangular areas contain the highly phagocytic IgM^+^ (upper) or IgM^−^ (lower) cell sub-populations (cells that have internalized a higher number of beads). Graphs showing the mean fluorescence intensity (MFI) of the internalized beads of IgM^+^ cells **(B)** or IgM- cells **(E)** (mean + SD; *n* = 6 fish). Graphs showing the ratio between the phagocytic IgM^+^ cells **(C)** or IgM^−^ cells **(F)** and the corresponding IgM^+^ or IgM- populations (mean + SD; *n* = 6 fish). Graphs showing the ratio between the highly IgM^+^ **(D)** or IgM^−^ **(G)** phagocytic cells and the corresponding phagocytic population in different conditions (mean + SD; *n* = 6 fish). Asterisks denote significantly different values between indicated groups (^*^p ≤ 0.05, ^**^p ≤ 0.01, and ^***^p ≤ 0.005).

In spleen, we observed that the pre-stimulation of IgM^+^ B cells with CpG ODNs significantly increased the percentage of phagocytic IgM^+^ B cells in leukocyte cultures when compared to either unstimulated cultures or cultures treated with non-CpG ODNs (Figures 5A,C). Along with this increase in the percentage of phagocytic cells, the mean fluorescence intensity (MFI) of internalized beads in splenic IgM^+^ B cells was significantly higher in CpG-treated cells compared to untreated cells (Figure 5B), indicating that the average number of particles internalized per IgM^+^ B cell was higher when IgM^+^ B cells were pre-stimulated. In concordance with this result, we found that the percentage of IgM^+^ B cells with a high number of ingested beads (highly phagocytic IgM^+^ B cells) was also higher in cultures treated with CpGs in comparison to untreated cultures (Figure 5D). Interestingly, in this case, the addition of non-CpG ODNs also increased the percentage of highly phagocytic IgM^+^ B cells when compared to untreated cultures (Figure 5D). When we analyzed the effects the CpGs had on the IgM^−^ population from spleen, no significant differences between the values obtained in CpG-treated cultures and those obtained in untreated samples were found (Figures 5E–G), although in some cases differences between the values obtained in CpG-treated cultures and those of non-CpG-treated cultures were observed (Figures 5E,G).

**Figure 6:**
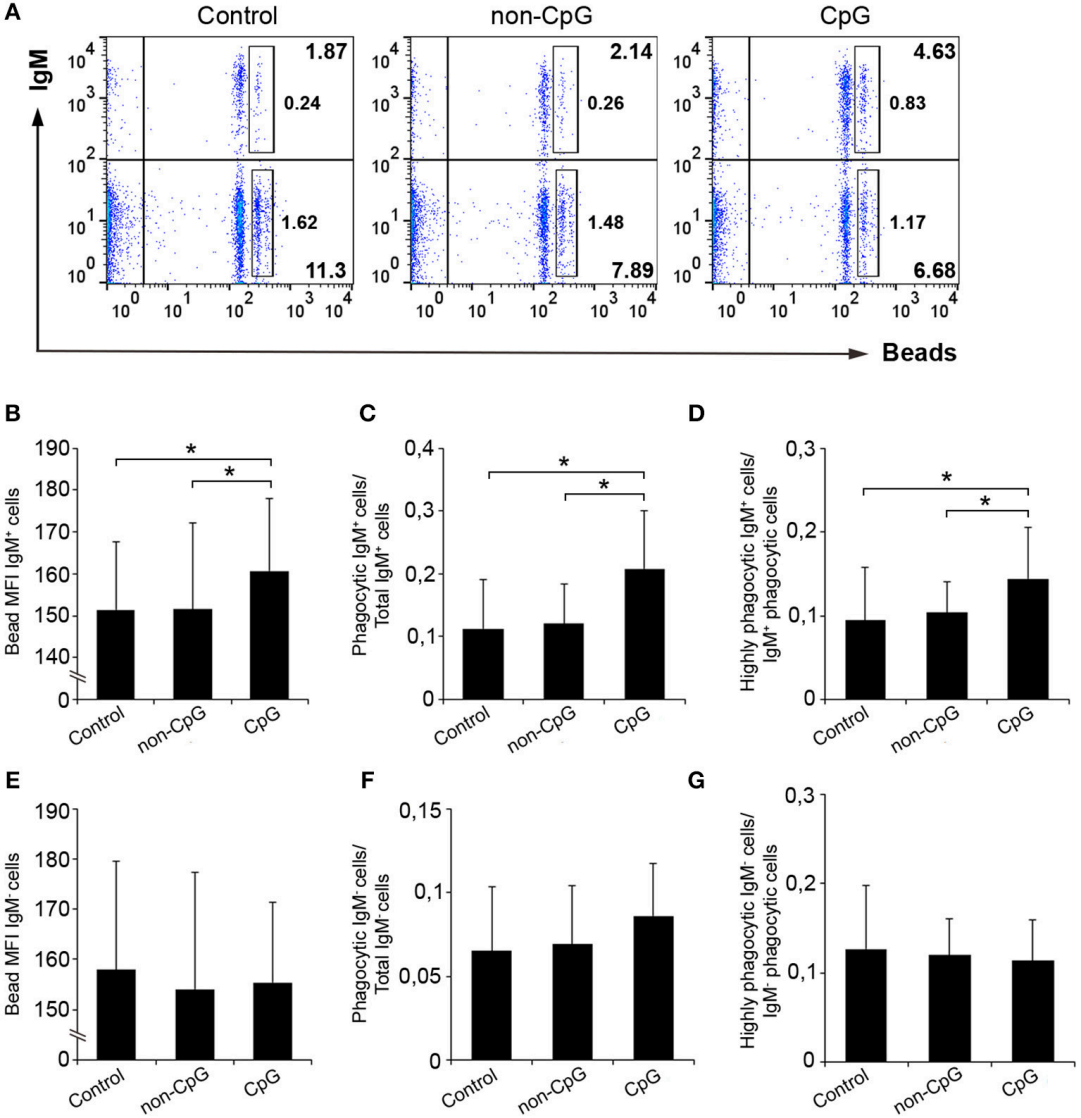
CpGs up-regulate the phagocytic capacities of IgM^+^ B cells from blood. Blood leukocytes were cultured in the presence of CpG, non-CpG, or media alone as previously indicated for 48 h at 20°C. After this time, cells were incubated with Crimson Red fluorescent beads and non-ingested beads removed as described in the legend of Figure 5. **(A)** Representative dot plots for each condition are shown. The rectangular areas contain the highly phagocytic IgM^+^ (upper) or IgM^−^ (lower) cell sub-populations (cells that have internalized a higher number of beads). Graphs showing the mean fluorescence intensity (MFI) of the internalized beads of IgM^+^ cells **(B)** or IgM- cells **(E)** (mean + SD; *n* = 6 fish). Graphs showing the ratio between the phagocytic IgM^+^ cells **(C)** or IgM^−^ cells **(F)** and the corresponding IgM^+^ or IgM- populations (mean + SD; *n* = 6). Graphs showing the ratio between the highly IgM^+^ **(D)** or IgM^−^ **(G)** phagocytic cells and the corresponding phagocytic population in different conditions (mean + SD; *n* = 6 fish). Asterisks denote significantly different values between indicated groups (^*^p ≤ 0.05.

In blood, the pre-incubation with CpGs also provoked a significant increase in the percentage of IgM^+^ B cells with phagocytic capacities (Figures 6A,C), in the number of ingested beads per cell (Figure 6B) and in the percentage of highly phagocytic cells (Figure 6D) when compared to both untreated cultures and cultures treated with non-CpG ODNs. No effects were exerted by CpGs on the phagocytic capacities of the IgM^−^ population in peripheral blood (Figures 6E–G).

### CpGs Synergize With BCR Cross-Linking in Blood IgM^+^ Cells

**Figure 7:**
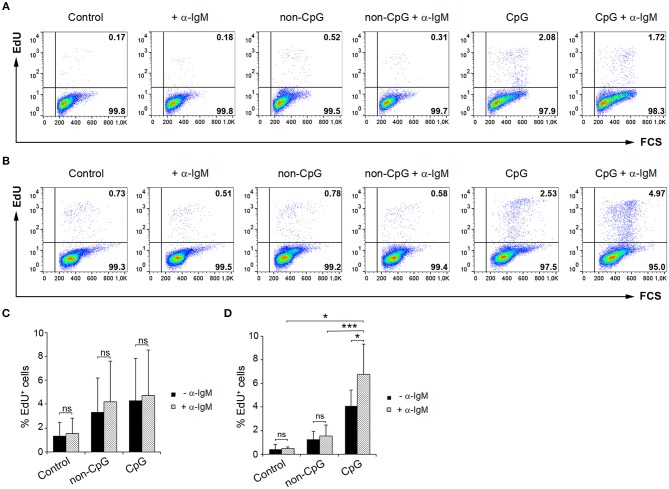
Synergistic effect of CpG and BCR cross-linking on B cell proliferation. Spleen and blood leukocytes were incubated with CpG (5 μM), non-CpG (5 μM), or media alone in the presence or absence of anti-IgM mAb (10 μg/ml) during 3 days at 20°C. After this time, cells were labeled with EdU (1 μM) and incubated for a further 24 h. The percentage of proliferating cells (EdU^+^) after the corresponding treatments was evaluated by flow cytometry. Representative dot plots from spleen **(A)** and blood **(B)** leukocytes for each condition are shown. Graphs show the percentage of EdU^+^ cells (mean + SD) among spleen **(C)** and blood **(D)** leukocytes (*n* = 5 fish). Asterisks denote significant differences between cells treated with CpG or non-CpG and/or anti-IgM and their corresponding controls (^*^p ≤ 0.05 and ^***^p ≤ 0.005).

Having established that CpGs induce the proliferation of IgM^+^ B cells on their own, we decided to analyze whether CpG could synergize with the BCR to induce a higher proliferation rate. To activate the BCR, we incubated the cells with anti-IgM as previously described (5). As reported before (5), the addition of anti-IgM alone was not sufficient to induce the proliferation of trout IgM^+^ B cells from spleen (Figures 7A,C) or blood (Figures 7B,D). However, when BCR cross-linking was combined with CpG stimulation, a proliferation rate higher than that observed in cultures stimulated with CpGs alone or with anti-IgM alone was observed in blood (Figures 7B,D). This synergistic effect although visible in some fish was not significant in spleen (Figures 7A,C).

## Discussion

CpGs have been proposed as promising adjuvants for fish vaccines (33). This presumed adjuvant activity has been ascribed on the basis of the results obtained in CpG-stimulation experiments performed mostly with total leukocyte populations in which cytokine gene expression, cell proliferation or oxygen or nitrogen radical production were measured [reviewed in Carrington and Secombes (34)]. Some of these experiments were carried out with macrophage-enriched populations or macrophage cell lines, thus the effects that CpGs exert on teleost macrophages are well-defined and include an increased antiviral activity, secretion of pro-inflammatory cytokines, and triggering of radical production [reviewed in Carrington and Secombes (34)]. However, the response of cells of the adaptive immune system to CpGs remains largely unknown in teleost. Very recently, Jenberie et al. (13) studied the effect of CpGs on IgM^+^ B cells from salmon, but the study was mostly based on analyzing the transcription of several immune genes in FACS isolated B cells stimulated or not with CpGs. Although that work confirmed that teleost B cells also react to CpGs, many aspects of how teleost B cells respond to these ODNs rested unexplored. Thus, in order to expand our knowledge on the effects that CpGs have on teleost IgM^+^ B cells, in the current study we have analyzed how CpGs modulate a wide range of immunological functions of IgM^+^ B cells, performing parallel experiments with splenic and blood populations.

As already described for different cytokines such as BAFF (35) or APRIL (36) and TLR ligands such as LPS (24), CpGs increased the survival of trout IgM^+^ B cells in cell culture. As occurred with LPS (24) or APRIL (36), this increased survival went along with strong proliferative effects on IgM^+^ B cells. In mammals, class B CpGs are particularly efficient in promoting the proliferation of B cells (4, 18, 19, 25, 37), however important differences in CpG-induced proliferation rates have been reported in different studies. Thus, for example, Bernasconi et al. (19) reported that human naïve B cells only proliferated in response to CpGs if the BCR was simultaneously activated, whereas in that study memory B cells were shown to proliferate in response to CpGs alone. On the contrary, other studies have reported high proliferation rates of naïve human B cells stimulated with CpGs alone (18, 25, 38). These differences might be due to the fact that in each of these studies, different CpG ODNs were used. Additionally, differences in source and activation state of the B cells used could also account for the differences observed. In our studies, we compared the effects of CpGs on both spleen (main secondary immune organ) and peripheral blood, but no significant differences were observed in what concerns the effects exerted by CpGs alone on survival or proliferation.

In mice, although the capacity of B1 cells to proliferate in response to CpGs is lower than that of murine B2 cells (4), only B1 and MZ cell populations are capable of differentiating to plasma cells in response to CpG stimulation in the absence of BCR cross-linking (4). This was demonstrated on the basis of augmented IgM secretion, increased CD138 expression and up-regulated transcription of plasma cell-specific markers such as Blimp1 and XBP-1 (4). In the case of rainbow trout, we have demonstrated that CpGs significantly increased the number of cells secreting IgM. As expected from B cells that were differentiating to plasmablast/plasma cells, an increase in size and a decrease in surface IgD and IgM expression were also evident. However, the fact that Blimp1 transcription is not significantly up-regulated in response to CpGs in isolated IgM^+^ B cells from CpG-treated cultures when compared to those of sorted IgM^+^ B cells from untreated cultures (data not shown) could be suggesting that CpGs are not able to achieve a complete differentiation of trout B cells to plasma cells. However, we have to take into account that Blimp1 has been designated as a protein required for the development of IgM-secreting cells and the maintenance of long-lived plasma cells in mammals (39), but not in fish. Hence, assuming that the requirements for B cell differentiation will be exactly the same in teleost fish is somehow risky, having established the great differences that exist between fish IgM^+^ B cells and mammalian B2 cells (5). Therefore, at this point, we can conclude that CpGs induce the differentiation of IgM^+^ B cells to antibody secreting cells (ASCs) as verified by increased IgM secretion, reduction of surface IgM and IgD expression and increased size, but whether these cells reach a full differentiation state should be further investigated. Intriguingly, when determining the effect of CpGs on the levels of surface IgM and IgD we found that non-CpG ODNs provoked a similar effect than that exerted by CpGs, suggesting that these nucleic acids are also recognized by immune cells and can activate the cells to some extent. In mice, non-CpG ODNs were shown to synergize with a specific antigen in stimulating specific B cells to proliferate, to express early activation markers and to activate the NF-κB pathway (40).

In addition to being responsible for the secretion of antibodies, B cells in both mammals and fish are professional antigen presenting cells capable of presenting to T cells antigens they acquire through the BCR in the context of MHC II (28). To investigate whether CpGs could affect antigen presenting capacities of trout B cells, we studied the levels of MHC II surface expression in stimulated and unstimulated cells. We found that IgM^+^ B cells from spleen up-regulated the levels of surface MHC II in response to CpGs. Similarly, human B cells treated with CpGs up-regulated MHC II and CD86 expression (18). In salmon, the up-regulation of MHC II in kidney IgM^+^ B cells treated with CpGs was demonstrated through Western blot (13). Interestingly, in trout, this effect was not exerted on blood IgM^+^ B cells when total leukocyte cultures were incubated with CpGs but were clearly visible when sorted blood IgM^+^ B cells were incubated with CpGs. Furthermore, both splenic and blood IgM^+^ B cells up-regulated the transcription of co-stimulatory molecules (CD83 and CD80/86) in response to CpGs. In salmon, stimulation of salmon kidney IgM^+^ B cells with CpGs alone increased the transcription of CD83 but not that of CD86 (13). In any case, whether the effects on MHC II surface expression are executed on the same population that differentiates toward a plasmablast/ plasma cell should be further explored, as it had been commonly accepted that as B cells differentiate to plasma cells MHC II surface levels decreased (41).

Fish B cells (32), similarly to mammalian B1 cells (31), have the capacity to phagocyte microparticles. This capacity has been related to a high microbicidal activity of fish B cells (32), as well as with a greater capacity to present particulate antigens they acquire through this mechanism (29). Of course this correlates with the fact that fish B cells have been shown to share many functional and phenotypic traits of mammalian B1 populations (5), implicated in the early innate response to pathogens. In this context, we thought of great relevance to determine if CpGs could affect an innate function of trout IgM^+^ B cells, such as their phagocytic capacity. Our results demonstrate that CpGs significantly up-regulated the phagocytic capacity of both splenic and blood IgM^+^ B cells. These effects of CpGs were evidenced by a higher percentage of B cells with phagocytic capacity, a higher number of beads ingested per cell and a higher percentage of cells with a high number of internalized beads. Interestingly, these effects were exclusively exerted on the IgM^+^ population both in spleen and blood, suggesting that phagocytic IgM^−^ cells do not respond to CpGs as IgM^+^ B cells in what refers to their phagocytic capacities. Although it may be possible different cell types are included within the IgM^−^ phagocytic population, IgT^+^ B cells should make up for most of these cells, given that IgT^+^ B cells have been described to account for ~12% of the lymphocyte population in spleen (10). Thus, the effect of CpGs on IgT^+^ B cells and whether CpGs affect the phagocytic capacities of mammalian B1 cells are interesting questions that should also be explored in the future.

The fact that human naïve B cells rapidly up-regulate TLR expression after BCR stimulation (19), strongly suggested that BCR signaling can synergize with TLR ligation. As expected, this synergy was demonstrated in human naïve B cells, but not in memory B cells that do not proliferate differently to CpGs when the BCR is simultaneously activated (19). In mice, this synergy between CpGs and BCR cross-linking was also visualized, with effects on both B cell proliferation and Ig secretion (37). In our experiments, we found that although a slight increase in the proliferative response of splenic IgM^+^ B cells was observed when anti-IgM and CpGs were combined, a significant synergy between these two signals was only observed in blood IgM^+^ B cells. These results demonstrate that CpGs have the capacity to amplify BCR-mediated signals also in teleost, but again point to important differences in the way B cell populations from different organs respond to CpGs.

In conclusion, our results show that CpGs have major effects on a wide range of adaptive and innate functions of IgM^+^ B cells from rainbow trout. These include lymphoproliferative effects and positive effects on cell survival. Additionally, experimental evidence point to CpGs provoking the differentiation of some of these IgM^+^ B cells to plasmablasts, although whether these cells reach a fully differentiated state or not remains undefined. CpGs were also shown to regulate the antigen presenting properties of IgM^+^ B cells and to amplify BCR-mediated signals, although in this case, significant differences in the way splenic and blood IgM^+^ B cells responded to the CpGs were found. Finally, CpGs were also shown to modulate innate functions of fish IgM^+^ B cells, such as their phagocytic capacity. All these results point to CpGs as excellent adjuvant candidates for novel vaccine formulation designs in aquaculture.

## Data Availability

All datasets generated for this study are included in the manuscript and/or the Supplementary Files.

## Author Contributions

RS performed most of the experimental work, with help from PD-R, EM, DM, and AG. CT and RS designed the experiments and wrote the main body of the paper, with contributions from PD-R and AG.

### Conflict of Interest Statement

The authors declare that the research was conducted in the absence of any commercial or financial relationships that could be construed as a potential conflict of interest.
