# Supplementary material for: CpG Oligodeoxynucleotides Modulate Innate and Adaptive Functions of IgM+ B Cells in Rainbow Trout
Source: Front Immunol. 2019 Mar 26;10:584. doi: 10.3389/fimmu.2019.00584 (PMC6443966; doi:10.3389/fimmu.2019.00584)
Supplement: Supplementary file 1 [file Data_Sheet_1.PDF]

Table S1. Real time PCR primers used in this study. Gene names, forward and reverse primer sequences and accession numbers are indicated.

Gene	Forward Primer	Reverse primer	Accession no.
**Actin**	TCCTTCCTCGGTATGGAGT	TTACGGATGTCCACGTCAC	NP_001117707.1
**TLR9**	TCTTCATAGAGCTGAAGAGGCCTCA	GTTCCCACTGAGGAGAAGTGTTTT	NM_001129991
**CD80/86**	GTGTTTCCTGGTTCTGGTATCTA	AACTTGCTGCTCCCTTTCCTC	FJ467621
**CD83**	GCTGTTGATAGCGGGAGGTA	TGTGGACTCAAGGCAATCTG	AY263793.1

Figure S1

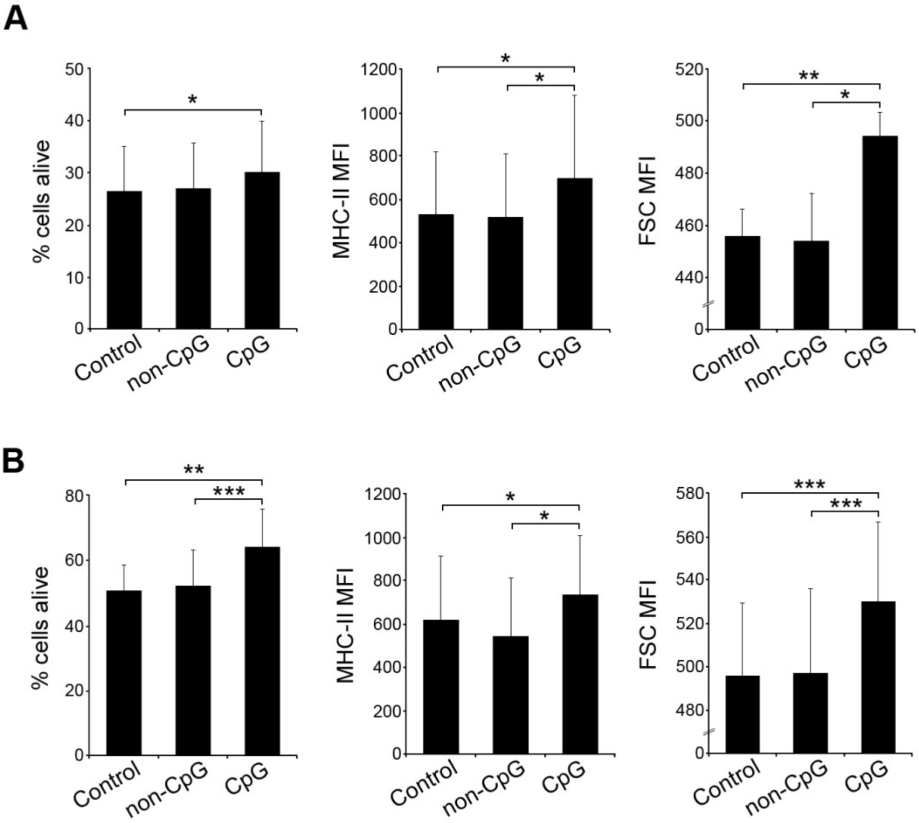

Figure S1. Incubation of sorted IgM$^+$ B cells with CpGs has stimulatory effects on cell survival, size and surface MHC-II expression. Sorted IgM$^+$ B cells were incubated with 5 µM CpG, 5 µM non-CpG or media alone for 3 days at 20ºC. Cells were then analyzed by flow cytometry to determine the percentage of cells that remained alive in the cultures, the levels of MHC-II surface expression and the size of the cells. Graphs showing cell survival, MHC-II MFI and FSC MFI values for IgM$^+$ B cells in spleen (A) and blood (B) are shown (mean + SD; n= 6 fish).
